# The Transactions of the American Medical Association

**Published:** 1861-11

**Authors:** 


ART. IV.—*The Transactions of the American Medical Association.* 1860. Vol. xiii. pp. 930.

IN looking over the thirteenth volume of the *Transactions*, our attention has been directed by a natural predilection to the papers read before the Surgical Section. We commenced reading with the anticipation of great pleasure, and ceased with the realization of great dissatisfaction. We felt deep regret that such productions, replete with bad English, vanity, and unfounded statements, should bear the impress of the Association. Our regret is more deeply felt when we consider that the volume is issued under the sanction of the body of the profession of our country, will be read by the representative men of science beyond the Atlantic, and will be placed in the libraries of medical associations in Europe as the exponent of American medical science in so far as it treats.

The first paper, entitled a "Report on the Various Surgical Operations for the Relief of Defective Vision," by MONTROSE A. PALLEN, M.D., of St. Louis, Missouri, is in itself exceedingly defective, treating of but a part of the operations, as those for anchyloblepharon, symblepharon, trichiasis, entropion, ectropion, and strabismus. It is true, a terminal note by the writer announces that for want of space the consideration of glaucoma, artificial pupil, and cataract will be deferred for another occasion. A poor excuse is better than none, unless it be, as in this case, so extremely poor that it is shadowless. Who limited the space? When appointed to report on the subject, was the writer informed that his paper must not exceed sixty pages?

Had the writer properly appreciated his duty and confined himself to a report on the various operations, instead of writing a treatise on the affections requiring these operations, sixty pages would have been ample space. In introducing the subject of strabismus, for instance, *seventeen* pages are devoted to the literature, etiology, and diagnosis of the disease, and *fourteen* to the operations, the writer stating that he finds it "absolutely necessary to consider the literature and pathology," and yet makes no use of such consideration in presenting the operations.

We do not dispute his assertion that he found such consideration necessary *for himself*, but we deem it quite unnecessary to have quoted page on page from the text books in reference to the diagnosis of strabismus, or matters of that kind, in order to report on the operations. It may be as well, however, that only so much did appear, for nothing new or noteworthy occurs in the "Report" thus far, and that in exceedingly bad taste. One would suppose the writer believed Mr. Macdonald the columbiad of ophthalmic literature, for he follows his miserable running glossary style.

It is indeed painful to read sentences so filled with brackets, French synonyms, and such like peculiarities, as for instance, "with the forceps, (*pinces à griffes;*)" and again, "an elevator, (*refouleurs,*)" etc., as if that made the matter more plain and intelligible. Such things in writing are, to say the least, in very bad taste.

As to the correctness of the use of the language in which it purports to be written, read on page 411. After stating that the patient was prepared for the operation for strabismus, he says: "The muscle was then divided with the scissors after the conjunctival wound was made, and it (the muscle) had been drawn out by means of a blunt hook." Now what would the reporter think of a surgeon, who, describing an operation, the amputation of a limb, for instance, should say, the tourniquet having been applied and the patient made ready, the bone was then divided with the saw after the incisions were made in the soft tissues, and it (the bone) exposed by means of a retractor?

Again, on page 412, describing the procedure of another surgeon in a like operation, is written, "a curved bistoury, (on its axis,) the cutting edge upon the concavity, the point small and heel broad, is then inserted under this cordiform fold and is made to cut its own way out through the muscle, tendon, fascia, etc. etc." Can a bistoury be curved other than upon its axis? Is *small* the opposite of *broad?* In operating for strabismus, would the surgeon cut both muscle and tendon? and after dividing the tendon, fascia, and one "*etc.*," where and what would the other "*etc.*" be?

In speaking of the procedure of Guérin in operating for strabismus, he says: "This plan is peculiar and ingenious: special instruments are necessary, which are three double hooks, (*érignes,*) a conjunctival perforator, (lance-shaped,) double edged and slightly curved on the flab; a Z-shaped myotome, concave on the back, convex on the edge, and elbowed, with its handle, (bayonet-shaped.)" All must fully understand so lucid a description as is thus presented. If a few more brackets had been interspersed, the reader would have been much assisted.

But such matters are of minor importance compared with practical points, and in consideration of them we pass to the concluding paragraphs of the paper in which they are all summed up, as follows:—

"As numerous and ingenious as are the various operations for strabismus, (many of which have been crowded out of this paper for the want of space,) it is surprising to see the number of successful attempts, and for this reason makes it the more difficult to determine as to the superiority of any one method.

"In order to decide as to the merits of the best operation, it is well to once more understand that strabismus is an alteration of the mean length of the opposing muscles, whatever may be the cause of that alteration, whether it be a change in the structure of the muscle itself or a real insertion of its tendon. After an operation for strabismus, generally, if not always, the attachment from cicatrization takes place posterior to the normal insertion; hence, to obtain a correction of the position and in proportion to the amount of deviation, the tendon must be divided farther back in such a ratio as will so induce the rectification.

"Thus, when the strabismus is but slight, the tendon should be divided as near the sclerotic as possible, and the wound in the conjunctiva should be but small, which must be united by a suture, and in proportion to the deviation, so will be the division from the sclerotic.

"The above remarks being premised and without an explanation which would not be out of place, I take the liberty of suggesting that the procedure of Mr. Critchett, *with the addition of a suture in the conjunctival wound*, is decidedly the best for such cases as present a less deviation than two lines from the central axis of vision; and that of Walton, superior to any of the others for such cases as present a greater deviation than two lines."

We here find several remarkable ideas presented, wherein grammar, physiology, mathematics, physics, and pathology are sacrificed. Let us query concerning them. Why should an operation be unsuccessful because ingenious? Does the writer mean the "merits of the best," or of all operations as to which may be the best? Is strabismus the alteration of the mean length of the opposing muscles? If of two muscles one be one and a half and the other one and a quarter inches in length, would not their mean length be one and three-eighths inches? and if one become contracted to one inch and the other relaxed to one and three-quarter inches, would their mean length be altered? Do we meet with but two causes of strabismus, one "a change in the structure of the muscle itself," and the other "a real insertion of its tendon?" What does the writer mean by *a real insertion* of a tendon? Do we ever have artificial insertion? Would a true, actual insertion of a tendon produce strabismus? On what system of philosophy is founded the idea that the eye is more freely liberated by dividing the tendon of the contracted muscle at a distance from its insertion proportionate to the extent, not "amount," of deviation? If an ass be tied to a post and the halter break near the head-stall, will not the animal escape just as freely as if it broke at the other end?

We see in the concluding sentence of the paper that the writer comes down to mathematical rules in applying to practice the procedures of Critchett and Walton. Now let us have the true mathematical guide in the application of his doctrine. Shall it be this?

If the deviation be but one line from the axis of vision, the tendon should be cut close to the sclerotic. If the deviation be two lines, then the tendon should be cut two lines from the point of insertion. If the deviation be three lines, then the tendon should be cut at the distance of four lines, and so on until we find the deviation of six lines, when the muscle should be divided ten-twelfths of an inch from its point of insertion. Why not?

One more query. What is the "*central* axis of vision"?

But enough. We are by no means pleased in reading a paper in nowise answering the anticipations of the subject.

The next paper in sequence is entitled a "Report on the Improvements in the Art and Science of Surgery in the last Fifty Years." By JOSEPH N. McDOWELL, M.D., of St. Louis, Missouri.

This paper is possessed of no qualities which entitle it to the considerations of a review or even notice. It is filled with inaccuracies, garrulity, vanity, and vulgarity.

We now come to the third and last paper, entitled a "Report on Morbus Coxarius or Hip Disease." By LEWIS A. SAYRE, M.D., of New York City. We consider this a peculiar title for a paper to be read before a scientific body. There is seemingly a design in this, for very few lay people seeing the paper in pamphlet form would understand the signification of morbus coxarius.

As this subject is one of great importance, and the purport of the paper of such a character, we have seen fit to examine more fully into it than into the previous ones. We have seen it stated in some notice of the report, that it is characterized by "directness, clearness, simplicity, and truth." We shall leave the reader to judge for himself whether such are its characteristics, after directing his attention to certain points, as follows:—

The writer introduces the subject by saying that it will be necessary for him to give a brief description of the most important anatomical structures entering into the composition of the hip-joint, in order that there may be a proper appreciation of the pathological views entertained in the report, and that the reader may fully comprehend the principles which he shall endeavor to establish as the proper basis of correct treatment.

There are but few diseases upon whose pathology much light is shed simply by descriptive anatomy, and here, in a malady whose pathology, symptoms, diagnosis, and treatment are based on such wide correlative circumstances, the writer gives but the plainest, most commonplace anatomy of the joint, at no time referring to the *anatomy of textures* which bears so important a part when considering the structures entering into the formation of all joints, in a pathological point of view. To give anything like an anatomical basis for pathological and therapeutical views as relates to hip disease, the whole apparatus of locomotion in the region should be described, together with the vascular system and nervous distribution of the parts.

From the anatomical introductory, we are led to the chapter on *causes*, in which the writer commits a graver error than that portion of the profession whose views in relation to the origin of the disease he positively condemns. Utterly discarding that well-grounded idea that morbus coxarius is of strumous origin, he affirms that, with but few exceptions, direct traumatic influence is the cause. The writer evidently draws no distinctive line between morbus coxarius and other affections of the hip-joint. But how does he prove his assertion, and attempt to disprove the established doctrine? Read the following:—

"Most practitioners will admit with me that they never succeeded in curing hip disease by an anti-scrofulous mode of treatment. To be sure the term scrofulous has essentially changed in its application within the past few years, and so has the treatment. Formerly, antimonials, the mercurials, and the iodine preparations and bark were in vogue—to-day, air, exercise, and generous diet are the chief remedies against this disease; but neither has cured morbus coxarius. Then derivatives were introduced extensively in order to eradicate the dyscrasic poison. The results have been equally unsatisfactory. In fact, whatever remedies were resorted to, morbus coxarius was never relieved thereby. It took its course to a spontaneous cure, or else to a fatal termination.

"Not until more attention had been paid to the *local treatment* of the disease have we had any satisfactory results. This fact alone should indicate the errors as to the nature of the disease formerly entertained. For it could hardly be asserted that a constitutional disease could be effectually relieved by merely local treatment. * * * * * * *

"And, again, experience teaches that no disease is capable of inflicting greater constitutional disturbances than affections of the bones or joints. Often have I seen phthisis of the lungs superadded to such cases in previously healthy subjects, etc.

"It seems, therefore, that the inevitable effects of the disease have been mistaken for its causes, that the attenuation of the patient, his anæmia, his vitiated nutrition in all respects, have been construed into previously existing scrofulous cachexia, originating and characterizing the disease; whereas they were consecutively developed by it, and were therefore results and not causes of the disease."

We deem these arguments extremely weak. So impressed with the non-strumous theory is the reporter that he admits but one construction and allows room to but one idea, and because means can be used to remove a certain amount of irritation, those means alone are curative; and because they relieve, we are all mistaken as to the nature of the disease. Equally ungenerous are his conceptions declared in the second quotation, himself completely transposing cause and effect, and admitting no exception, because he has seen phthisis supervene on hip disease.

Read now the following as to the examination required to ascertain the cause of any given case:—

"It requires generally a very close examination to find out these causes, since the disease does not usually immediately follow the injury, but often commences or manifests itself weeks and even months after the accident, so that the patient and his friends naturally forget the accident and its connection with the disease, until specially reminded of it in the investigation."

If, now, we take this groundwork for our doctrine, then we must bow to the anti-strumous theory. Most cases of morbus coxarius occur in children. Would it be possible to find a child that had not fallen or met with some injury within any given number of weeks? Now, if morbus coxarius become developed, the injury was the cause, according to the reporter. It seems that he, in his ardor to support the theory, makes no distinction between vital and exciting causes. The cachexia exists, we believe, and but awaits an opportunity for exhibition afforded by exposure, injury, or otherwise.

The *symptoms* are next discussed, and the opening sentence corroborates our declaration that the author of the report draws no distinctive line between morbus coxarius and other affections of the hip-joint, for he says: "The symptoms of this disease vary according to the different stages in which we find it, and the *character of the inflammation*, whether it be acute or chronic, *rheumatic*, *strumous*, or *purely traumatic*." The italics are our own.

In proceeding to give the symptoms, he makes a division according to the three stages of the disease. Passing without comment over the first division, we enter upon an examination of some of the points under the head of the—

"SECOND STAGE.—This inflammation, unless arrested, will readily produce effusion, and if this be of much amount, it will produce a *peculiar* deformity, viz., an *apparent* elongation of the limb; *eversion* and abduction; flattening of the natis; the rima natis on the diseased side being lower than on the sound side; flexion of the thigh on the pelvis, and the leg slightly flexed upon the thigh. If the effusion be excessive, or the inflammation acute, you will have an *apparent* anchylosis, caused by muscular contraction, which is an involuntary act," etc. * * * * * * * *

"The flexor muscles of the thigh, the tensor vaginæ femoris, the pectineus, and rectus femoris, are so firmly contracted that the whole pelvis moves on the opposite acetabulum," etc. * * * * * *

"This rigidity does not depend upon true or bony anchylosis; for by division of the flexor and adductor muscles, or by puncture of the joint, you will have free motion of the limb, showing that there has been no bony anchylosis.

"That the mere presence of liquid in the perfectly closed joint is capable of producing, and therefore *accounting for the said immobility*, is clearly demonstrated," etc.

Here we find an anomalous condition of things. We have effusion producing all the deformity and marked superficial symptoms, and also flexing the leg on the thigh, and the thigh on the pelvis; and, if excessive, even an apparent anchylosis, which is *also* caused by muscular contraction. We, moreover, find the individual muscles named which produce this seeming anchylosis by their firm contraction. Let us see what the associated action of these muscles is. They *flex* and *adduct*, and, putting on firm contraction, flex the thigh firmly or completely upon the body. Then, how do they produce immobility, acting in only one general direction? And when these muscles which *flex* and *adduct* put on such firm contraction, how is it we get *apparent elongation*, *eversion*, and *abduction?* But let us go a little further; we may be informed. It is next declared, as we see above, that this immobility can be relieved by a division of the flexor and adductor muscles. We do not see any smoother sailing here. Why should the *adductor magnus*, *longus*, and *brevis* with the *gracilis*, be cut, when the *pectineus* alone of this group is contracted, and the division of the rectus and tensor vaginæ femoris, which are contracted, be neglected? It is declared that these muscles produce, by their contraction, this apparent anchylosis, and yet the concluding paragraph of the last quotation asserts that it is clearly demonstrated, *that the mere presence of liquid in the perfectly closed joint is capable of producing, and therefore of accounting for the said immobility.* This whole matter seems to be considered in a very careless manner, totally regardless of connection, giving no evidence of appreciation of the true state of the case.

Under the *third stage*, in speaking of the escape of the effusion by rupture of the capsule, it is written that "this change from the second into the third stage is sudden when the opening in the joint (query, *joint* or *capsule?*) is large," etc., or gradual, if the opening be small or fissure-like. I have seen the former take place in a night, and the latter require weeks until the change was accomplished. Here we have that all-sufficient cause of all the symptoms of the second stage, effecting by its escape a most wonderful change. But it is less wonderful than the superior insight of the reporter into the workings of nature as disturbed by this disease. Through the thick surroundings of the hip-joint he sees with ease the magnitude and form of the opening through the capsular ligament, and marks the time of rupture, though the result be not effected for weeks. All other causes for the change of deformity are drowned in *effusion.* The cause of the deformity, according to the writer of the report, depends "upon muscular contraction and twisting of the pelvis." We will contrast the bearings of this remark with previous quotations and the following partial table of symptoms found on page 477.

| Second Stage. | Third Stage. |
|---|---|
| Limb (apparently) longer. | Limb (apparently) shorter. |
| " abducted. | " adducted. |
| " everted. | " inverted. |
| " flexed in both joints. | " flexed in hip-joint only. |

It will be remembered that, in speaking of the deformity of the second stage and the symptoms as here tabulated, the writer of the report says that effusion is the cause, and that there is an immobility produced by muscular contraction. In the third stage we have immobility from muscular contraction and an exactly opposite deformity. If the effusion produces all the symptoms of the second stage, how is it that as soon as the effusion escapes, the limb, instead of assuming its previous position, passes into a directly opposite one? In the second stage, according to the author of the report, the flexor muscles of the thigh, the tensor vaginæ femoris, the pectineus, and rectus femoris are the muscles implicated, giving eversion and abduction contrary to the known action of these muscles. The deformity of the third stage is directly opposite and is produced by muscular contraction. Now, if the afore-named muscles produce the abduction and eversion, what muscles produce the adduction and inversion? Though so definite in naming the muscles so firmly contracted in the second stage, no mention is made of the agents of the deformity in the third stage.

The remainder of the chapter is devoted to the labor of demonstrating the theory, that dislocation never occurs in the disease under consideration. Having predestined his mind by the following declaration, "the peculiar change that takes place, in many instances, upon the rupture of the capsule, in all the symptoms, and the suddenness of the occurrence have led to the *false idea* that a *luxation had taken place*," the writer, in his enthusiasm to support his idea, confounds heads with shafts, and, because a capsular ligament remained unruptured, declares that luxation had not taken place, though the head of the femur rested on the dorsum ilii, as here quoted:—

"*The head of the femur was lodged on the dorsum of the ilium.* The capsular ligament and synovial membrane were much dilated, and at the superior part their *attachment to the bone was thrust upwards*, so that, *although the head of the femur was no longer in the acetabulum, it was still within the cavity of the joint.*

"Here we have the testimony of Sir Benjamin Brodie, that '*the head of the femur was lodged on the dorsum of the ilium*,' and in almost the next sentence, he says, '*it was still within the cavity of the joint.*' Comment, it seems to me, is unnecessary, for this cannot be called luxation, according to the ordinary definition of that term."

And, again, on the same page, referring to Mr. Skey, we read, "the left femur was dislocated on the dorsum ilii," etc., and, stating that an operation for its removal was determined upon, he quotes, as regards the condition or actual state as observed at the time of operating, as follows: "The head of the femur had been entirely absorbed," etc. The reporter then says: "I would simply ask how it was possible that there could have been a dislocation on the dorsum ilii, if the head of the femur be entirely absorbed? Can a bone be luxated when it has no existence?"

Now let us simply ask, in the first place, must a capsular ligament be ruptured to perfect a luxation? If called to see a patient with all the symptoms of a dislocation present, would the writer cut to ascertain if the capsular ligament were ruptured before pronouncing it a dislocation? Mr. Skey says "the femur was dislocated," and the head absorbed. Does the femur disappear on the absorption of its head? Does it not still exist? If the head be absorbed, does the femur remain in its normal relation, distance, etc., with the innominatum, or is it dislocated for lack of support? If the writer hopes, by such evidences, to sustain his views and stands to so explicit a definition of luxation, he would do well to observe greater care in his own expressions than to say as he does, "instead of a luxation of the hip, we have in fact," mark the words, "a luxation, or rather a displacement of the acetabulum itself."

In bringing the consideration of the *third stage* of symptoms to a close, the writer describes and gives an illustration of a specimen for which he acknowledges his obligation to Dr. Carnochan. The specimen we well recollect, having seen and examined it when it was presented before the N. Y. Medico-Chirurgical College. We remember, too, the discussion which arose, some averring it to be the result of other than hip disease, Dr. Carnochan himself, who presented it, being unable to give any history of the case. In reference to this case, the reporter goes on to state, as follows:—

"This was a case of hip disease of nearly three years' standing, the result of an injury in a previously good constitution, and the disease progressing so insidiously that the patient did not apply for treatment to any surgeon until it had arrived at its third stage, when it was mistaken for dislocation, and so diagnosticated by all who saw it; and yet the *post-mortem* revealed the fact that the capsular ligament had never been ruptured, but still contained the head of the femur which had never been out of it.

"This case was not brought to the city until he was almost in a moribund condition, and then the gentleman who saw him, and who is one of the best surgeons in this country, mistook it for a luxation upon the dorsum ilii, and presumed, from the careless manner in which the history of the case was detailed, that it had occurred at the time of the accident more than two years before, and as it was a hopeless case nothing was proposed for it. I never saw the case until after death, when, upon making careful inquiries among his friends, I found that the limb had been in its present shortened position only a few months, but previous to that it was as they 'thought much longer and more crooked in the knee, and a great deal more painful, and since his leg had become shorter he was much easier of his pain, but his constitution run down so fast they feared he would die,' and therefore brought him to the city for relief."

On the 28th of March, 1861, at a meeting of the N. Y. Medico-Chirurgical College, Dr. Carnochan called attention to the fact that he, at a previous meeting, presented a specimen exhibiting the ravages of disease in the hip-joint; and, moreover, that the case was wholly involved in obscurity, as he stated at that time, and yet, in a pamphlet he held in his hand, entitled a "Report on Morbus Coxarius," by Dr. Lewis A. Sayre, reprinted from the *Transactions* of the Amer. Med. Association, the writer had given details and particulars of the case which he was wholly unable to account for, inasmuch as the specimen was taken from the "Dead-house" at the State Emigrants' Hospital under his own charge, and no history accompanied the case.

Under the chapter on *diagnosis*, the report presents some valuable differential facts in tabulated form taken from a lecture of Dr. Bauer. Other than this, the remarks are meagre, being confined to a consideration of the differential symptoms of morbus coxarius and sacro-iliac disease.

We have thus reached the chapter on *treatment*, on the examination of which we enter with some hesitation on account of the disregard of the reporter for physical laws, physiology and pathology.

We find Dr. Sayre dividing the treatment, according to the stages of the disease, into three parts, and, commencing with the first, he says: "But the most important of all, and upon which all prospect of success will depend, is rest of the joint," to which we all without doubt give assent. Before leaving the consideration of the first stage of treatment, forgetful that motion and rest are antagonistic in their natures, he says that he was led to contrive some plan to remove pressure from the synovial membrane, "and, at the same time, permit motion of the joint, thereby retaining the capsular ligament in a healthy condition, *for motion is just as essential to a joint as light is to the eye;* it is *in fact its physiological function,*" etc., with the most careless lack of consideration of the difference between a healthy joint and a diseased joint, or a healthy eye and one with retinitis. Does the reporter treat inflammatory affections of the eye, by keeping the patient in a light room, or reading by gas-light, because it is the "physiological function?"

In the *second stage* the reporter makes use of the same means as in the first stage, but seemingly for a different purpose; why, we have yet to learn. After giving some directions as to the local treatment, he advises slight but permanent extension "to relieve the pressure from the ligamentum teres." In the first stage he used the same means to remove "the pressure from the synovial membrane." Our knowledge of physics would lead us to the conclusion that the more we drew from the ligamentum teres the more pressure it would suffer. And when we consider, too, that the cavity of the joint is filled and the capsule distended with fluid, extension certainly but increases the pressure upon both the synovial membrane and ligamentum teres. It is rest that relieves the symptoms, and not extension.

Passing in further consideration of the treatment of this stage of the disease, the writer presents us with something new. In speaking of puncturing the joint to evacuate the contents, he says: "In removing this intolerable hydrostatic pressure, I simply imitate nature," etc.; and again having entered upon the third stage of treatment, referring to the same matter, he says: "In the former the lymph escapes into the cellular tissue, spontaneously relieving the hydraulic pressure of the joint," etc. Here we have a new feature in physics. Hydrostatics and hydraulics in a peculiar relation to pathology! A new and singular application of two words, of entirely different signification, to describe one condition, and both words so inappropriate, it seems hardly credible that they could have been used, seeing that their signification is utterly contrary to the idea desired to be conveyed.

The writer's grammar is equally defective in other places. Read this: "The general attitude was partially recumbent on the right side and semiflexed." The reporter should bear in mind that "attitude" is an expression of posture indicative of some emotion, and that, when used in connection with "recumbency," it is totally at variance with a proper use of the English language. A patient so much cast down and crippled with morbus coxarius as in the case he describes, would make a sorry picture *attitudinizing*.

On page 523 we read, "placed into the well-lined wire breeches." It was not our intention to say anything about this apparatus, and we should not, had the writer preserved a deference for the rules of the English language. "The" is decidedly definite, and as we have seen nothing as yet in reference to an apparatus that was "*well lined*," we suppose something new is to be presented. The "wire breeches" of Dr. Bauer are as different from the apparatus of Dr. Bonnet, of Lyons, as the straight splint is from the double inclined plane, although the reporter refers to them as being one and the same thing. What "the well-lined wire breeches" are we do not know.

Such matters are, however, of slight importance, indicative only of the carelessness with which the Report has been written. Of much greater importance are the following, which we find uttered while discussing the exsection of the head of the femur, and its results:—

"According to my personal observation, the intermediate substance is formed of very firm fibrous structure, which in a comparatively short time acquires the firmness of whalebone, or at any rate a sufficient mechanical strength for the support of the patient.

"The lower extremity of this intermediate substance is invariably and intimately connected with the femur, and the more closely so if the periosteum has been saved at the time of the operation; it is also connected with the surrounding soft parts, whereby it acquires additional strength; but is not *directly* attached to the ilium, but seems to form an artificial articulation.

"*Having had no opportunity of a post-mortem examination of an articulation of this kind in the hip, I am unable to give a detailed description of its anatomical construction.*"

The italics are our own.

Here we see the exact condition of the parts, the anatomical character, and the points of attachment and freedom of the new material, given with the confidence of an anatomist after having made a hundred post-mortem examinations, and, as he says, according to his personal observations; and yet the next sentence declares that he never observed it, for he never made a dissection of an articulation of this kind in the hip.

We have but one more matter of the kind to present before we proceed to review the mechanical treatment of morbus coxarius, as presented in the paper. Speaking, at page 532, of an operation he performed for the removal of the head of the femur, and stating that there was a retention of matter, he says:—

"And in order that it might have a free escape, I therefore with strong forceps divided the ilium and ischium at the outer border of the acetabulum, and the ischium and pubis at the inner, and cut out the *entire plane* of the ischium almost to the tuberosity; it was entirely denuded of periosteum on both sides, and only had attachment at the insertion of the ligaments. I also removed the upper and outer border of the acetabulum, and almost the entire body of the pelvis."

Here we have another instance of the writer's unbounded expression. What is the "entire plane" of the ischium? Why, after removing so much, did he also take away "almost the entire body of the pelvis?" Were both innominate bones diseased? Was it double morbus coxarius? Will not the reader conclude that the matter had pretty "free escape" after such an operation?

We have now to consider the *mechanical* features of the treatment, concerning which we, with many others, give acquiescence to a certain extent; but not to the exclusion of all other means. On page 488 we find the first mention of instrumental or mechanical appliance, by reference to the wire apparatus of Dr. Bonnet, the pulley and weight of Sir B. Brodie, the straight splint of Dr. March, and, in the reporter's words, "the instrument I have devised." Continuing, on the same page, we read: "It was in consequence of this accident occurring in several instances that I was led to contrive some plan," etc. On the next page: "for a full description of my instrument;" and again, on page 513, "treated without division of muscles with my splint;" and in like manner in various places mention is made of the instrument. On page 495 we find the following: "I only suggest to assist nature in effecting this spontaneous cure by mechanical appliances;" and again, on page 500, we read: "and at the same time, the superiority of the plan I have suggested."

These quotations carry to the mind of the reader but one inference, and that is, that the writer of the report first suggested the treatment and devised the instrument, for he positively declares it. Let us see for a moment what this instrument is, and who devised it. To do this, let us turn to page 499 where the details of a case commence, in the reading of which we arrive at a seeming solution. On page 507 we read a description of the instrument which is, as we have seen, called his splint; and we find it to consist of a steel strip, with means of attachment for extending and counter-extending bands, the latter of which is of rubber tubing. The writer goes on to give the directions as to the proper manner of applying the instrument, the style of applying the plaster, bandage, etc. But preceding it all, we find a plate introduced, under which is printed "H. G. Davis's Splint for Hip Disease, as manufactured by Otto & Reyndors, 58 Chatham Street, N. Y., since 1855," five years ago, accompanied by a full description, and the proper manner of applying it, and the style of applying the bandages, plaster, etc. The one is just like the other; there is not a single difference in any essential point, and all the directions, as given by the author of the Report, are the same as those of the inventor of the original instrument, from whom the reporter learned the use and application of the instrument, as noticed in the quotation on the next page. Seeing this, we are led to examine a little more closely, and we find, on page 505, that in November, 1859, a patient came under the care of the reporter, and he desired to make extension of and still keep up motion in the affected limb, but was unable to do it. How he succeeded at last will be seen by the following:—

"He returned to the city under my care in November, 1859, much improved, but with imperfect motion, and I observed that he always had more or less pain for one or two days after the motion had been applied, which I thought was owing to the fact that I could not keep up *sufficient extension while I was applying the motion.*

"This was a desideratum I had tried long to accomplish, but never succeeded to my satisfaction until Dr. H. G. Davis, of this city, applied to him one of his instruments, which answered the purpose admirably, and in its construction embraced the very principles I had so long sought to obtain.

"As Dr. Davis is, I believe, the first person who has constructed an instrument embracing these important advantages—extension with motion—I have taken the liberty of presenting a plate of the same, and his own remarks in regard to its method of application. I have made, as I think, some very important improvements and modifications of this instrument, which I will describe more fully hereafter."

After the description of Dr. Davis's instrument we find the following:—

"This instrument of Dr. Davis was applied in this case with the happiest results for a few days, but it soon began to excoriate the groin, and the method of extension was not satisfactory, and could not be controlled at will, but would be either too feeble or too severe, and I therefore constructed an instrument embracing all the principles in this, but much more effectual in its application, more comfortable to the patient, and entirely under the control of the surgeon."

We see here that the reporter found that there were certain matters connected with the instrument which he thought could be improved, and the first thing was the perineal band, and he therefore made use of India-rubber tubing, which he declares will not excoriate the groin, as follows:

"This catgut is attached at either end of the perineal or counter-extending belt, which is made of thick India-rubber tubing, and, being firmly secured at either end, makes an elastic and comfortable air cushion for the perineum, and will not excoriate or chafe the parts."

And again—

"I find by the use of the India-rubber tubular air cushion, there is no danger of excoriating the groin."

As far as the India-rubber tubing goes, and the possibility of its acting as an air cushion, there is a great deal of fallacy. The moment the tubing is put upon the stretch and pressed against the perineum it becomes collapsed upon itself, and acts as a plain flat band of rubber, than which there is nothing more productive of excoriation. Facts are stubborn things, and chafe sometimes worse than perineal bands of any make or material.

But enough of this. The experience is scarcely more marvelous than that indicated in the next quotation.

"The perineum becomes accustomed to the pressure in a few days if it is not too severely applied at first, and the patients begin to run around with ease and comfort, after which it is impossible to persuade them to abandon it, even for some time after they are entirely cured. At least this has been my experience."

Let us examine into this a little. The first use of the instrument dates December 3d, 1859. The Report was presented to the American Medical Association on the 7th of June 1860, that is, just about six months after. The quotation indicates plainly that within six months, and indeed a much shorter space of time, morbus coxarius is "entirely cured." He says plainly enough that such has been his experience.

The temptation is strong for us to query a little here and make still further contrasts, but we forbear; and, leaving this part of the Report, undertake an examination of the concluding chapter on exsection of the head of the femur, as presented under the head of "*Remarks*" on page 534.

Giving a history of the operation more or less correctly, he mentions certain cases as regards dates and the success or fatal results, and then says:—

"In this country this operation seems to have attracted but little attention until I published my first case in the *New York Journ. of Med.* for January, 1855, and which is condensed in this Report, (No. 38.) This was the first successful case operated upon in this country, and I thought at the time, the first time the operation had been performed here; but I afterward learned that Dr. Bigelow, of Boston, had performed it a year before, but had not published the case. Dr. Bigelow's case terminated fatally on the twelfth day; mine is therefore the first successful case recorded in this country." * * *

"I have heard that Dr. Parkman, of Boston, exsected this bone in 1853, but have been unable to obtain any particulars of the case."

Refer now to the table and see Nos. 32 and 33, where both cases are given, and this very case of Dr. Parkman reported with "particulars" as a "perfect recovery." How can the reporter's operation in 1854 be "the first successful case recorded in this country," when, by referring to the *American Journal of the Medical Sciences*, Dr. Parkman's case will be found recorded, as also Dr. Bigelow's—one in the July number, 1852, and the other in the April number, 1854?

Proceeding, we find the following on page 536:—

"By a careful examination of all the fatal cases, and the cause of death as far as given, I have been unable to trace a single instance in which the operation could be ascribed as a cause of death."

Mark the words—"I have been unable to trace a single instance," etc., and yet read the following:—

"*Textor's Second Case.*—Gangrene of the wound took place, and the patient died on the fourth day.

"*Hawkins's Case.*—Died on the third day; although his patient was very much exhausted, she suffered but very little from the operation, and was much relieved and improved for two days.

"*Roux's Case.*—Died on the seventh day from secondary hemorrhage, the rough section of the femur producing slough of the femoral artery.

"*Kinlock's Case.*—Died in thirty hours. No particulars given." (Kinlock himself says, in the *Charleston Medical Journal*, May, 1857, that reaction never properly took place.)

"*Esmarch's Case.*—Died on ninth day, of pyemia.

"*Langenbeck's Case.*—On the eighth day, of phlebitis and metastatic abscess.

"*Fock's First Case.*—On the thirteenth day, of pyemia.

"*Fock's Third Case.*—Died on fourteenth day, of phlebitis and metastatic abscess.

"*Hancock's Case.*—Did well for eight days, when erysipelas supervened, of which she died.

"*Kuhn's Case.*—Died on the sixth day; but the caries involved also the sacrum and ischium."

We are not aware what the reporter considers to be the necessary concomitants to make an operation the cause of death. The cases as quoted from the Report certainly seem to have been hurried on to dissolution by the operation. If the author of the Report requires the patient to die on the table to mark the operation as fatal, well and good for him, perhaps, but not acceptable to us. Most certainly death following thus speedily and from causes as quoted, produced by the operation, must bear as adversely in its application to exsection of the head of the femur as if in relation with any other operation.

We now turn to a consideration of the "table," concerning which, on page 535, the reporter says as follows:—

"In preparing the following table regard was had to exsection in morbus coxarius only, as it is in reference to this particular class of cases that we wished to estimate the value of the operation."

In an examination of the cases it is not our intention to detail all the errors, but simply to call attention to the more prominent points of an erroneous nature, they being sufficient to characterize the Report for unreliability.

In the first place, Case 1 is a case of spontaneous separation, by the reporter's own showing. So is Case 41, (see "Lehre von den Blutigen Operationen," etc., von B. Gunther, 1856, p. 203,) and such will be seen is the character of Case 69, by reference to authority. Such cases, the author of the Report says, do not belong to exsections, and on that account he puts aside the cases of Kluge, Batchelder, Vogel, Schlichting, and others. It will be seen, too, that Cases 4, (see Lond. Lancet, Am. Rep., vol. vii. p. 384,) 6, (see ditto,) and 11, (see Arch. Gen. de Med., 4me Serie t. xv. p. 549,) were not for morbus coxarius. Case 4 was a case of fracture; Case 6 was a case of caries of the trochanter; and Case 11 was an operation by M. Maisonneuve for anchylosis, after the manner of Barton.

Chronic rheumatism certainly cannot be called morbus coxarius, and under such a classification we must place 23, 57, and 97, as will be seen by referring to the authorities whence they are derived.

We have therefore to cut off *nine* cases at one sweep. Is this all? Let us see. Upon further examination, we find Case 18 is a repetition of Case 9; that Case 16 is none other than Case 15; that 52 is repeated in Case 65, and that 60 and 89 are one and the same. In plain terms, *four* cases are thus made to count *eight*.

This diminishes the total of cases tabulated by *thirteen*, and instead of 110 cases there are left but 97. It would seem almost a pity to cut off any more, but 25, 50, 51, 90, and 93 are presented without authority, and, therefore, in performing our operation of exsection, we find it necessary to gouge out more dead matter and thus remove *five* more cases, and so leave the total at 92 instead of 110 cases.

Our whole purpose is effected by the preceding; but we desire to call attention to a few other points in error, and, in conclusion, modify the "Recapitulation." By reference to the table great variability is seen in the tabulation, some cases being given in full, while others are presented in such an imperfect manner that we should be justified in cutting them off in a thorough criticism. Thus, for instance, Case 19 is presented with only the sex, result, year, and name of the operator, without any authority, while the table is arranged for *nine* facts. Gathering around us our cloak of patience, and observing other points, we find in Case "2" *fourteen* given as the age, when the patient was but eight years old. In Case "17" we see it reported as "wound closed well," and that he *recovered*, when the real case was, "has to a great extent closed up;" and, instead of recovery, *death* followed three and a half months after. Case 12 is reported as having died *two and a half years* after the operation of exsection. The truth is, that death took place in *sixteen months*. Case 35 is presented thus: "*Rapid improvement; suppuration profuse;*" when in the authority it reads: "*On the nineteenth day gradual improvement.*" "*Suppuration small.*" We must attribute such things to carelessness or imperfect investigation, and where so many points of error are found, our faith in whatever else appears is proportionately weakened.

In the "Recapitulation" we find as follows:—

| | |
|---|---|
| Total number of resections of head of femur for caries . . | 110 |
| Died . . . . . . . . . . . . | 36 |
| Reported as unfavorable . . . . . . . | 2 |
| Recovered, with more or less useful joint . . . | 72 |
| | 110 |

Our limited investigation diminishes the total by 18 cases, leaving the sum at 92. Of these 18 cases, 14 are reported as successful, and 4 as having died. Instead then of 72 successful cases, we have only 58; and instead of 36 cases of death, there were 34. Is this the sum total of the errors to be found? By no means. Does the "Recapitulation" tally well with facts in other respects? Let us see for a moment. In giving results, the reporter dates death in some of the unsuccessful cases as far even as a year and more, and very properly, too; but he puts under the head of recoveries cases under treatment at the time of reporting, some of them not more than ten days, and concerning which nothing definite was known. We would enumerate 36, 37, 45, 49, 58, 66, 68, 75, 84, 101, 104, 105, 106 as cases of this character, and therefore not with any confidence to be quoted in favor of exsection; nor to the contrary, though with much more correctness. This, then, diminishes the total of recoveries by 13, thus making the number stand only 45. But we must not stop here. By glancing over the cases and their authorities, we find 17, as before mentioned, reported as recovered. The patient died. (See *Lancet*, Aug. 14, 1852.) We find Case 59 likewise reported, but the patient died. (See *Lancet*, Oct. 17, 1857.) Case 70 stands in the same category, for the patient died in about three months. (See *Lancet*, Oct. 10, 1857, and April 28, 1860; *Med. Times and Gaz.*, Oct. 24, 1857.) The same is true of other cases, some of which are known to have died, though not reported. Taking, however, only such cases as are here authenticated, we diminish the recovery cases by three, and to the same extent add to the unsuccessful cases, together with the other errors, making quite a different summing up. As follows:—

| | | |
|---|---|---|
| Total cases | | 92 |
| Successful | 42 | |
| Died | 37 | |
| Under treatment, and therefore doubtful | 13 | |
| | 92 | |

To contrast the features of the reporter's "Recapitulation" with the summing up of our analysis, we find, rejecting fractions, a difference of 21 per cent. against the operation, setting aside entirely the unfavorable cases. In plain words, the reporter gives about 65 per cent. of recoveries, while we find but about 44 per cent. The reporter gives but *two* cases as unfavorable. We find *thirteen* of the same nature, and consequently, while the reporter's 65 per cent. is weakened by but *two* cases, our 44 per cent. is similarly affected by *thirteen*, really diminishing the per cent. of success.

The profession well knows the disposition of its members to make a good appearance for their operations and cases, and the failure of many to report the results, especially if not favorable; and, therefore, unless positive report as to result is recorded, we more correctly estimate the result as unfavorable in all operations of a grave character.

We thus have reached the end of the matter under consideration, and, although we have made but a limited investigation, we think sufficient has been discovered to cause us to repeat our regret that such productions should be presented under the auspices of the American Medical Association.

